# Effects of muscle fatigue on directional coordination of fingertip forces during precision grip

**DOI:** 10.1371/journal.pone.0208740

**Published:** 2018-12-10

**Authors:** Wenjing Hu, Na Wei, Zong-Ming Li, Ke Li

**Affiliations:** 1 School of Control Science and Engineering, Shandong University, Jinan, China; 2 Department of Geriatrics, Qilu Hospital, Shandong University, Jinan, China; 3 Department of Biomedical Engineering, Cleveland Clinic, Cleveland, OH, United States of America; 4 Suzhou Institute of Shandong University, Suzhou, China; Manchester Metropolitan University - Cheshire Campus, UNITED KINGDOM

## Abstract

Object manipulation requires well-coordinated force vectors involving both magnitudes and directions. Despite extensive studies about force magnitudes during manipulation, relatively little is known how the muscle fatigue could affect the directional coordination of fingertip forces. This study aims to examine the effects of muscle fatigue on inter-digit coordination of force directions during precision grip. Sixteen female subjects performed precision grip with their thumb and index finger before and after fatigue tasks, which required subjects to produce continuous submaximal pinch strength on the apparatus for a duration more than 200 s. Both their left and right hands were evaluated using the same testing protocol. The means and standard deviations of the coordination angle and the projection angle were applied to quantify the directional coordination across the digits and the force vector direction of each individual digit. Results showed that fatigue led to significant reduction in the mean values of coordination angle and that of projection angle of the index finger in the ipsilateral hand (*p* < 0.05). Meanwhile, fatigue induced increases in both the standard deviations of coordination angle and projection angle of both digits in the ipsilateral hand (*p* < 0.05). These results imply that the muscle fatigue could interfere with the grasping stability by altering the directional coordination of all the involved digits and the control of force directions for each individual digit. These findings provide insights into fatigue-related changes of force directional regulation and coordination in dexterous manipulation.

## Introduction

Muscle fatigue is manifested by exercised-induced transient reduction in the capacity of muscle to produce force. Limited contraction of muscle fibers [1, 2], reduced intensity of nerve signals [3], altered motor unit recruitment [4, 5], decreased maximal force production [2, 4] and increased force fluctuation [4, 6] are usually associated with muscle fatigue. An intriguing issue is how the muscle fatigue affects the motor coordination for goal-directed fine motor skills, such as grasping and manipulating an object.

Mechanically, stably grasping and holding an object are subject to a variety of constraints. For example, the vertical shear force offsets the gravity [7], the ratio of tangential to normal forces should be smaller than the coefficient of friction [8, 9], the moments applied upon the object satisfy equilibrium condition [10, 11]. To meet all these mechanical constraints, the digits need to produce appropriate force vectors according to the objects’ physical properties (e.g. center of mass, friction condition), movement status (e.g. in acceleration, in rotation) and task demands (e.g. lifting, descending) [10, 12, 13]. The force vectors generated by different digits should be well coordinated to achieve suitable overall effects. During precision grip, for example, the force directions together with force magnitudes exerted by the thumb and index finger can prevent the held object from unexpected rotation and slipping [14]. The individual force vector deviates from the direction perpendicular to the contact surface, and the resultant force vector of the thumb and index finger are not aligned in opposition, generating a vertical net force to overcome the object’s weight and inertia [15, 16]. Reductions in grip force and increases in tangential forces resulting from directional alterations in force vectors can lead to a higher risk of grasping failure [17, 18].

Previous studies have reported that the directional coordination of digit force vectors during precision grip may change with age [19] and neuromuscular conditions [17]. As performing a precision grip with thumb and index finger for a key-slot task, the old adults exhibited greater variance of the digit-tip force angles in both frontal and horizontal plans than the young adults [19]. In the post-stroke individuals with hemiparesis, the force vector of the paretic digits had deviations from the direction perpendicular to the grip surface more than twice as much as for the asymptomatic digits; and the paretic thumb had about 18% greater deviation of grip force direction than the paretic index finger [17]. These findings suggest that the directional coordination of digit forces during precision grip is indicative of the status of the neuromuscular function. Previous studies have also reported that the altered muscle activations associated with muscle fatigue may lead to changes in grip force control, including decreased grip force [20], decreased grip force to load force ratio [21] and decreased correlation between grip force and load force [7]. However, it remains unclear whether the directional coordination of the thumb and index finger forces during precision grip is modulated by muscle fatigue.

The purpose of this study was to examine the effects of muscle fatigue on directional coordination of force vectors of the thumb and index finger during precision grip. The hand muscle fatigue was realized by a high-level sustained submaximal voluntary contraction, before and after which a precision grip was performed. We hypothesized that fatigue would result in more deviated force vectors of each individual digit with respect to the contact surface, and a lower angle across the force vectors of the thumb and index finger.

## Materials and methods

### Subjects

Sixteen right-handed female subjects (age: 21.94 ± 1.98 years; height: 162.94 ± 3.68 cm; weight: 53.63 ± 6.95 kg) enrolled in the experiment. All participants were strongly right handed (the Edinburgh handedness inventory scores were 91.25±6.41) [22]. All subjects had normal or corrected-to-normal vision, without history of musculoskeletal injuries on their upper extremity, neurological disorders or cognitive dysfunction. Each subject gave an informed consent prior to the experiment. The experimental procedures were approved by the Institutional Review Board of Shandong University and were in accordance with the Declaration of Helsinki.

### Experimental setup

**Fig 1:**
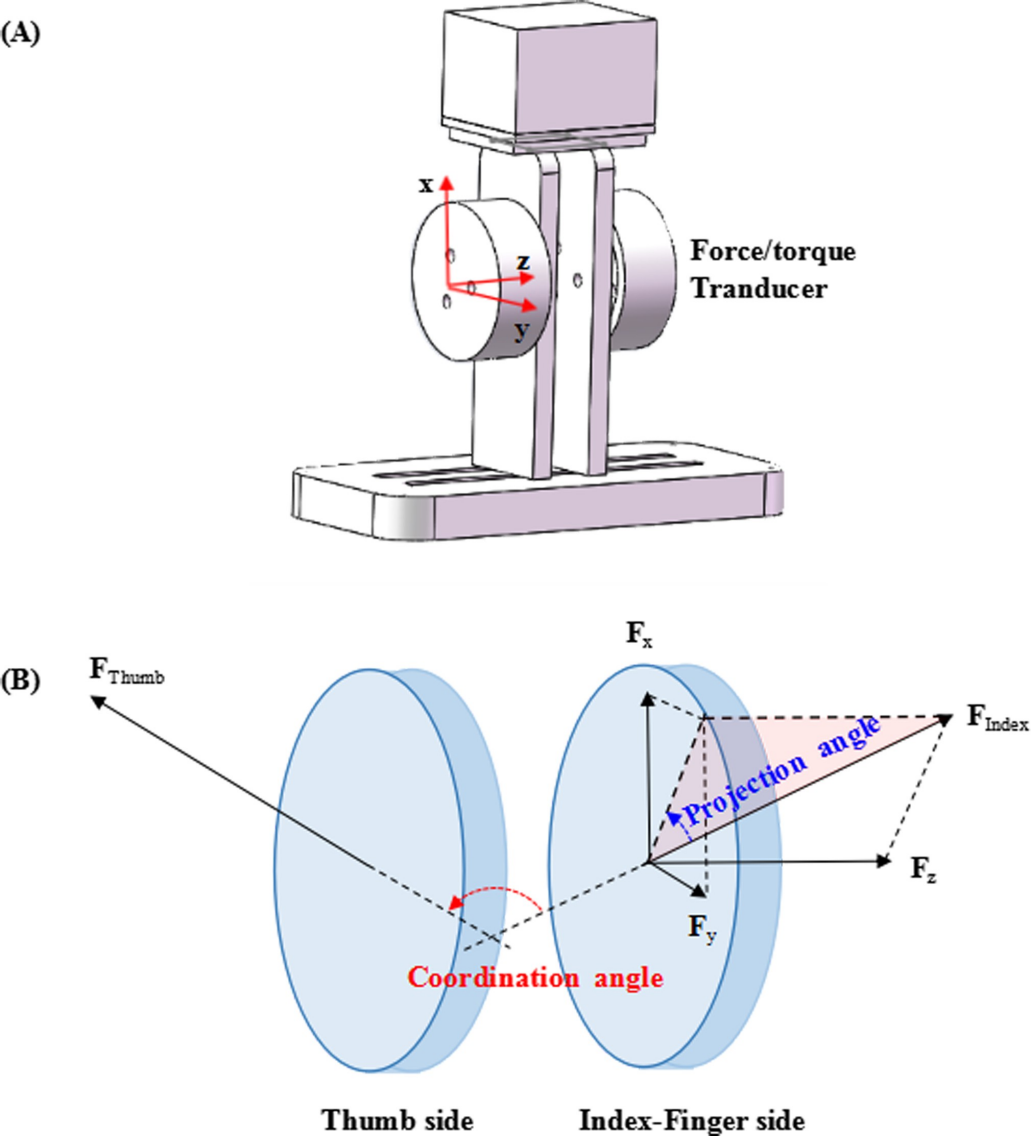
The apparatus and parameter definition. (A) A custom-designed apparatus consists of two force/torque transducers. The common coordinate system is described in the red color; (B) The definition of coordination angle and projection angle.

Digit forces were measured by 2 six-component force/torque transducers (Nano 17, ATI Industrial Automation, Inc., Apex, NC) mounted on a custom-made apparatus (Fig 1A). The grip surfaces were oriented in parallel with a pinch span of 50 mm and were covered with 100-grit sandpaper to increase the coefficient of friction. The gross weight of the instrumented apparatus was 172 g. The ranges of each transducer were 0 ~ 50 N for both the *x*- and *y*-axis and 0 ~ 70 N for the *z*-axis. The sensitivity of each transducer was 0.0125 N for all the three directions (*x*-, *y*- and *z*-axis). The transducers were mounted on the handle by precisely positioning that the *x*-axis and *y*-axis were along the vertical and horizontal directions in the contact surface of each transducer, and the *z*-axis was in the perpendicular direction to the contact surface (Fig 1A). Data collection was implemented using a custom Labview program (National Instrument, Austin, TX). Force signals were recorded at a sampling frequency of 1000 Hz. The data of the experiment were saved on a public repository (Figshare, DOI: 10.6084/m9.figshare.7188377. URL: https://figshare.com/articles/Dataset_zip/7188377).

### Experimental procedures

**Fig 2:**
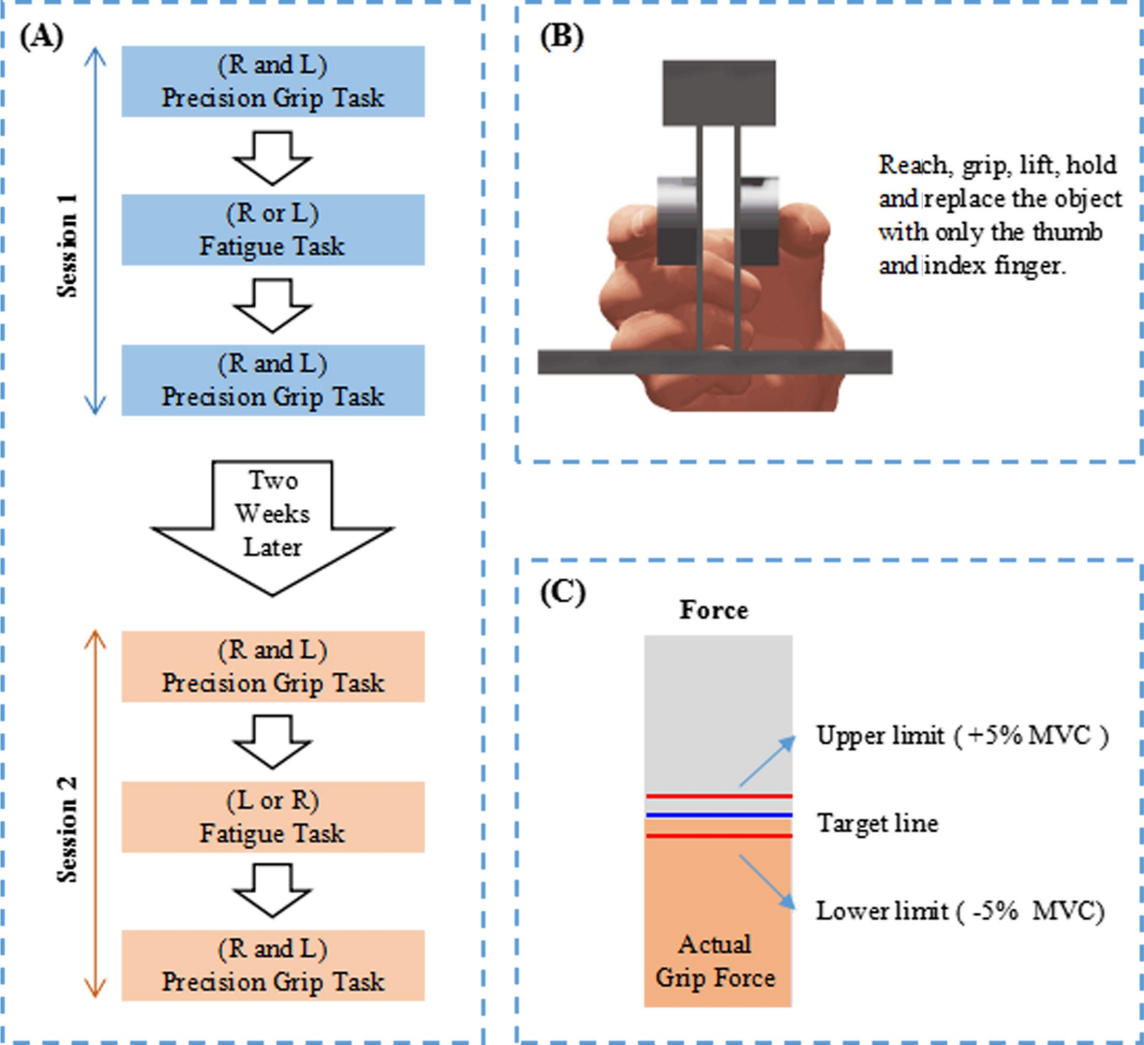
The experimental protocol (A), precision grip performance (B) and visual feedback for fatigue task (C).

The experiment consisted of two sessions, with a two-week rest between sessions (Fig 2A). In each session, subjects performed precision grip tasks following the same protocol before and after a fatigue task that could effectively evoke a muscle fatigue. Within a session, the precision grip tasks were performed by both the left and right hands, whereas the muscle fatigue was realized only on one side–either left or right hand. The difference between the two sessions was the fatigue hand selected. Within a session, the precision grip tasks were performed by both the left and right hands, whereas the muscle fatigue was realized only on one side–either left or right hand. The difference between the two sessions was the fatigue hand selected. Two randomizations were set up to prevent potential interference in results. First, the testing order for fatigue task was randomized. For example, some subjects received a right fatigue in session 1, followed by a left fatigue in session 2; whereas the others had an inverse order, receiving fatigue on the left hand in session 1 and on the right hand in session 2. Second, testing order for precision grip task was randomized. For example, under any of the fatigue conditions, some subjects performed precision grip by their right hand first, followed by their left hand; by contrast, the others had an inverse sequence, with their left hand first, followed by their right hand.

At the beginning of each trial, the apparatus was positioned at 30 cm from the edge of testing table, in alignment with the shoulder of the grasping hand. For the precision grip task, subjects were instructed to use their thumb and index finger to contact the center of the grip surfaces, lift the apparatus about 5 cm above the testing table, and hold the apparatus as stably as they could for 50 s (Fig 2B). Subjects were required to maintain the base of the apparatus parallel to the testing table without obvious tilt, using a minimum grip force to prevent the apparatus from slipping. When time was out, the apparatus was replaced on the testing table.

The precision grip task was repeated 3 times on the left and right hands, respectively. Each subject was familiarized with the testing protocol before the formal experiment. Before the fatigue task, maximal voluntary contractions (MVCs) of both hands were tested following a standard protocol [23]. Considering all participants were female subjects whose MVCs are usually far less than the upper-limit of Nano 17 transducer (70 N), the MVCs were measured using the same apparatus used for precision grip. Subjects were encouraged to use their maximal effort to pinch upon the center of the contact surface of the apparatus three times. The average normal forces of three trials were calculated as the MVC values.

For the fatigue task, subjects were instructed to continuously exert their pinch force with the thumb and index finger at a certain level of maximal voluntary contraction (MVC) for 200 s. A target line (the blue line in Fig 2C) representing 50% MVC was show on a screen. The actual pinch force, which was the mean force of the thumb and index finger, was demonstrated as the yellow bar (Fig 2C) on the screen. Subjects were instructed to pinch the apparatus and control the yellow bar to match the target line. A range within ±5% MVC around the target line, shown by red lines in the screen (Fig 2C), was allowed for the force fluctuations during the sustained fatigue contraction. The fatigue task was performed three trials. After each fatigue trial, the precision grip was performed immediately on the left and right hands following the protocol as described above. Considering the effects of fatigue were accumulating, the target line for the fatigue task was reduced to 40% MVC for the second trials and 30% MVC for the third trial, in order to guarantee the successful performance of the fatigue task.

### Data processing

The three-directional force components of the thumb and index finger during precision grip were recorded before and after fatigue generation. For each trial, signals at the first and the last few seconds were excluded, so that only the middle part (10–40 s) of the signals were left for further analyzed. Following the previous studies, a coordination angle and a projection angle were used as metrics to assess the inter-digit force vector coordination [15]. Briefly, the coordination angle was defined as the angle formed by the thumb and index finger vectors (Fig 1B). The coordination angle was computed as follows:

$$coordination\;angle=\cos^{-1}\left[\left(\vec{F_{T}}\times \vec{F_{I}}\right)/\left(\left|\vec{F_{T}}\right|•\left|\vec{F_{I}}\right|\right)\right]$$ (1)

where the $\vec{F_{T}}$ and $\vec{F_{I}}$ are the 3-D force vectors of the thumb and index finger, respectively. For both the thumb and index finger, the projection angle was defined as the angle of the force vector with respect to the *x*-*y* (shear) plane and was calculated as:

$$projection\;angle=\tan^{-1}\left(\left|F_{z}\right|/\sqrt{F_{x}{}^{2}+F_{y}{}^{2}}\right)$$ (2)

where the *F*_x_, *F*_*y*_ and *F*_*z*_ are digit force components in *x-*, *y-* and *z-* axis, respectively. Following the formula (2), the projection angle of the thumb and that of the index finger were calculated, respectively (Fig 1B). The coordination angle and projection angle were first proposed in the study of precision pinch with increasing and decreasing force levels [15], followed by the studies on compliant pinch [24] and on the effects of carpal tunnel syndrome [25]. Both amount and variability of coordination angle and projection angle could be able to reflect inter-digit force directional coordination. The higher projection angle, for example, suggest higher normal-to-shear force ratio, implying the grasping has a higher safety margin; and the higher coordination angle would suggest higher normal-to-shear force ratios of each digit and thus a more efficient force coordination across digits. The parameters were calculated using MATLAB 2015a (The Mathworks, Natick, MA, USA).

### Statistical analysis

Statistical analyses were performed using SPSS 23.0 (SPSS Inc., Chicago, IL). All the results were presented for the left and right hands separately. There were totally three fatigue conditions: (1) non-fatigue, which means neither the left nor the right hand underwent fatigue; (2) left-fatigue, which means only the left but not the right hand was in fatigue; and (3) right-fatigue, meaning only the right but not the left hand was in fatigue. For each condition, the grip force (*F*_*z*_) as well as the means and standard deviations of coordination angle and projection angle were calculated as parameters for the following statistical analyses. The means and standard deviations of coordination angle and of projection angle were at first examined for normality using a Kolmogorove-Smirnov test (K-S test). A one-way repeated measures ANOVA was employed to examine the differences of the means and standard deviations of coordination angle among the three conditions (non-fatigue, left-fatigue and right-fatigue) for either the left or the right hand. Two-way repeated measures ANOVAs were employed to examine the differences of the mean and standard deviation of projection angle across the three conditions (non-fatigue, left-fatigue and right-fatigue) and the two digits (thumb vs. index finger), for either the left or the right hand. The Huynh-Feldt correction was used when the assumption of sphericity was violated. Post-hoc pairwise comparisons were performed between the three fatigue conditions using a Holm-Sidak test followed by a pairwise *t*-test. The hand (left vs. right) was not a factor for any of the statistical analyses. Therefore, no comparison was performed between the left and right hands. A *p*-value of less than 0.05 was considered statistically significant.

## Results

**Fig 3:**
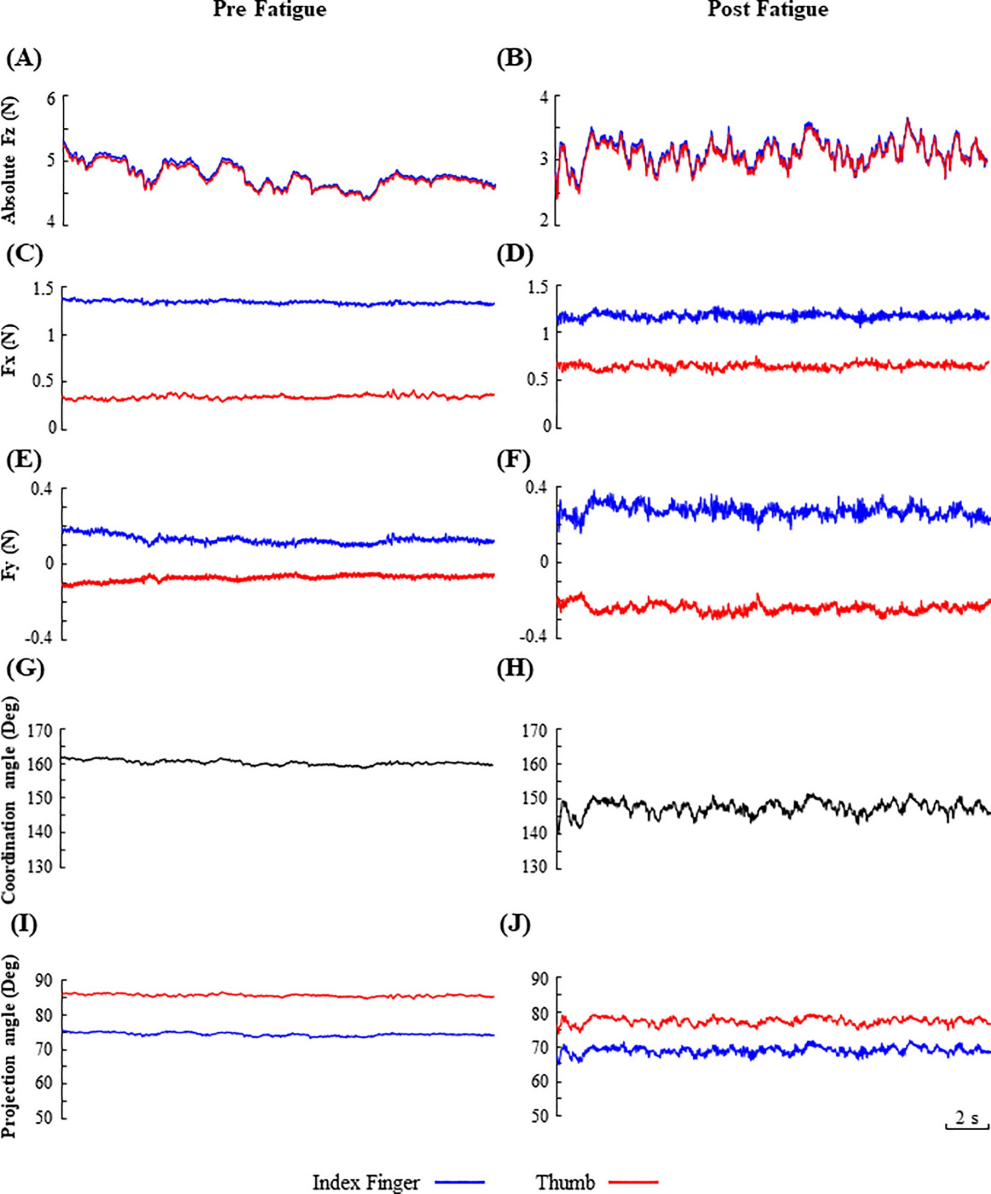
Force vector components of the thumb and index finger and the time course of coordination angle and projection angle before and after left fatigue from a representative subject. (A)—(F): the absolute values of *F*_*z*_, the *F*_*x*_ and the *F*_*y*_ before and after fatigue; (G)—(H): coordination angle of two digit force vectors; (I)–(J): projection angle of the thumb and index finger forces.

The MVCs were 39.70 ± 5.05 N for the right and 38.03 ± 4.56 N for the left hand. The force components in the *x-*, *y-*, and *z-* axis during one trial of left-hand precision grip from one representative subject are depicted in Fig 3. The left columns demonstrate the force components without fatigue and the right columns demonstrate the trials after the left fatigue. Compared with the non-fatigue condition, the *F*_*z*_ under left fatigue condition shows relatively lower absolute values but high fluctuations (Fig 3A and 3B); and the *F*_*x*_ and *F*_*y*_ after left fatigue present higher fluctuations than their counterparts under the non-fatigue condition (Fig 3C–3F). No significant difference of the grip force (*F*_*z*_) was found between fatigue conditions (Table 1). In addition, the coordination angle and projection angle of the same trials were shown in Fig 3G–3J. The coordination angles across the two digits and the projection angles of the thumb and index finger after left fatigue shows relatively lower values but higher fluctuations than their counterparts under the non-fatigue condition.

**Table 1:** Means and standard deviations of grip forces during precision grip.

Parameters	Fatigue conditions	Left hand		Fatigue conditions	Right hand	
		Thumb	Index finger		Thumb	Index finger
**Mean**	**Non-fatigue**	3.72 ± 1.81	3.80 ± 1.82	**Non-fatigue**	3.70 ± 1.32	3.57 ± 1.28
	**Left-fatigue**	3.11±1.33	3.11 ± 1.34	**Right-fatigue**	3.38 ± 1.25	3.22 ± 1.21
**Standard deviation**	**Non-fatigue**	0.21± 0.14	0.21± 0.14	**Non-fatigue**	0.18 ± 0.07	0.19 ± 0.09
	**Left-fatigue**	0.27 ± 0.20	0.27 ± 0.20	**Right-fatigue**	0.22 ± 0.11	0.21 ± 0.11

Data were presented as mean ± standard deviation.

**Fig 4:**
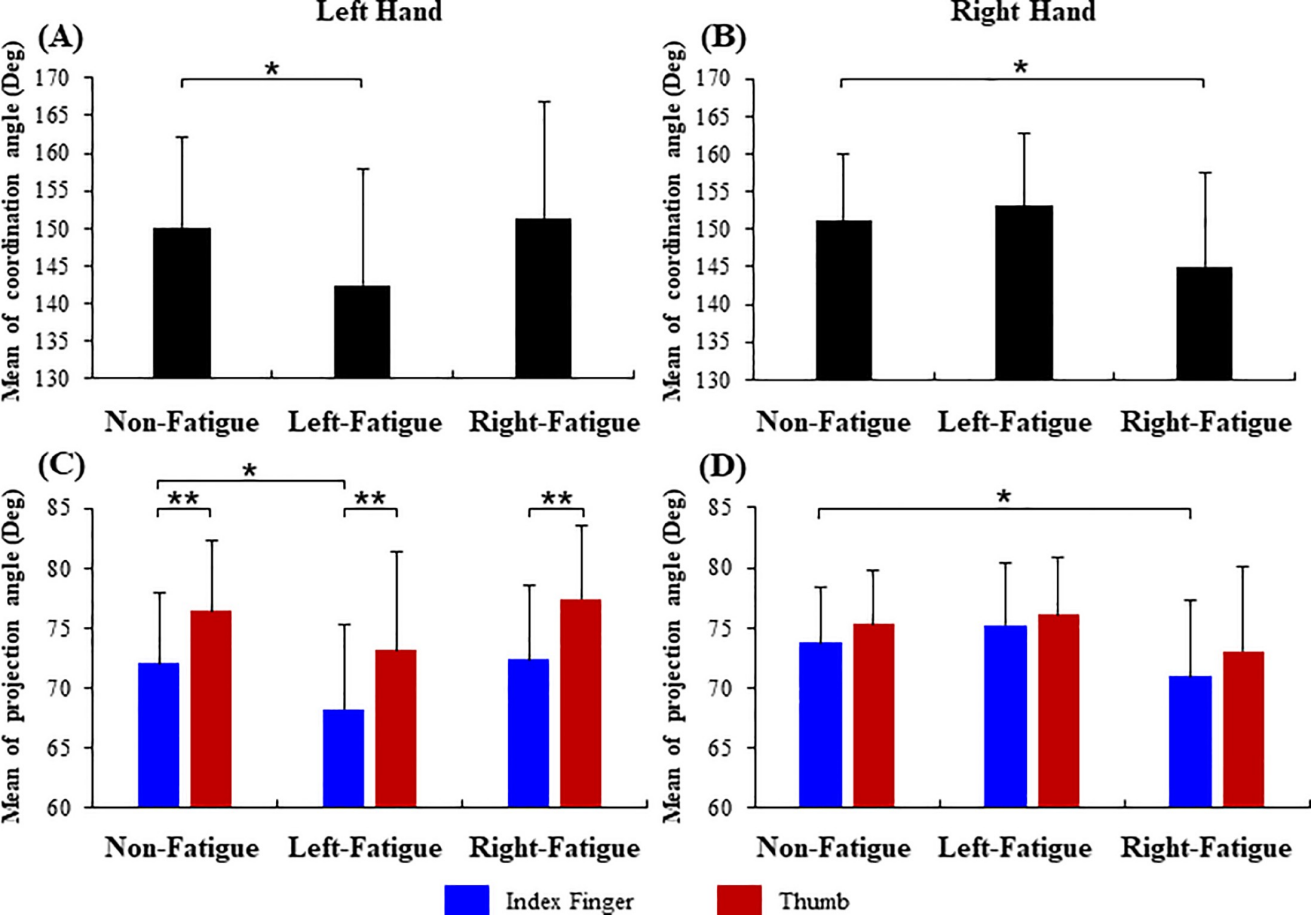
**Mean of the coordination angle and projection angle under non-fatigue, left-fatigue and right-fatigue.** (A) and (B) mean of the coordination angle for the left and right hands, respectively; (C) and (D) mean of the projection angle for the left and right hands, respectively. * *p* < 0.05; ** *p* < 0.001.

The mean of coordination angle under the non-fatigue, left-fatigue and right-fatigue conditions are shown in Fig 4A and 4B. The ANOVA tests showed significant main effects of fatigue conditions on the ipsilateral precision grip performance for both the left (*F*_1.19, 17.82_ = 5.21, *p* = 0.03, Fig 4A) and right hands (*F*_1.35, 20.83_ = 5.97, *p =* 0.02, Fig 4B). Specifically, for the left hand, the coordination angle under the left-fatigue condition was 142.49 ± 15.43°, significantly lower than the coordination angle under the non-fatigue condition (150.13 ± 12.02°, *t* = 2.28, *p* = 0.04); for the right hand, the coordination angle under the right-fatigue condition was 145.03 ± 12.59°, significantly lower than the condition without fatigue (coordination angle: 151.19 ± 8.75°, *t* = 2.28, *p* = 0.04). The fatigue did not affect the contralateral coordination angle performance, considering no significant difference was observed between the right-fatigue and non-fatigue conditions for the left-hand coordination angle (*p* = 0.34) and between the left-fatigue and non-fatigue conditions for the right-hand coordination angle (*p* = 0.19).

The mean of projection angle under the non-fatigue, left-fatigue and right-fatigue conditions are shown in Fig 4C–4D. The ANOVA tests showed significant main effects of fatigue conditions on the ipsilateral projection angle control during left- (*F*_1.18, 17.64_ = 4.55, *p* = 0.04, Fig 4C) and right-hand precision grip (*F*_1.30, 19.43_ = 4.84, *p* = 0.03, Fig 4D). In addition, significant main effects of digit (thumb vs. index finger) on the projection angle were only observed in the left hand (*F*_1,15_ = 59.82, *p* < 0.01) but not in the right hand (*p* = 0.10). No significant interaction was found between the fatigue conditions (non-fatigue, left-fatigue and right-fatigue) and the digits (thumb and index finger) for the both hands. For the left hand, the projection angle of the index finger under left-fatigue condition was 68.24 ± 7.05°, significantly lower than that under the non-fatigue condition (72.04 ± 5.89°, *t* = -2.26, *p =* 0.04); for the right hand, the projection angle of the index finger under the right-fatigue condition was 70.99 ± 6.26°, significantly lower than that under the non-fatigue condition (projection angle: 73.76 ± 4.59°, *t* = -2.28, *p* = 0.04). No significant difference was observed in the index-finger projection angle between the right-fatigue and non-fatigue conditions for the left hand (*p* = 0.66) and between the left-fatigue and non-fatigue conditions for the right hand (*p* = 0.05). Furthermore, no significant difference was observed in the projection angle of the thumb across the three fatigue conditions during either the left- (*p* = 0.05) or right-hand precision grip (*p* = 0.12).

**Fig 5:**
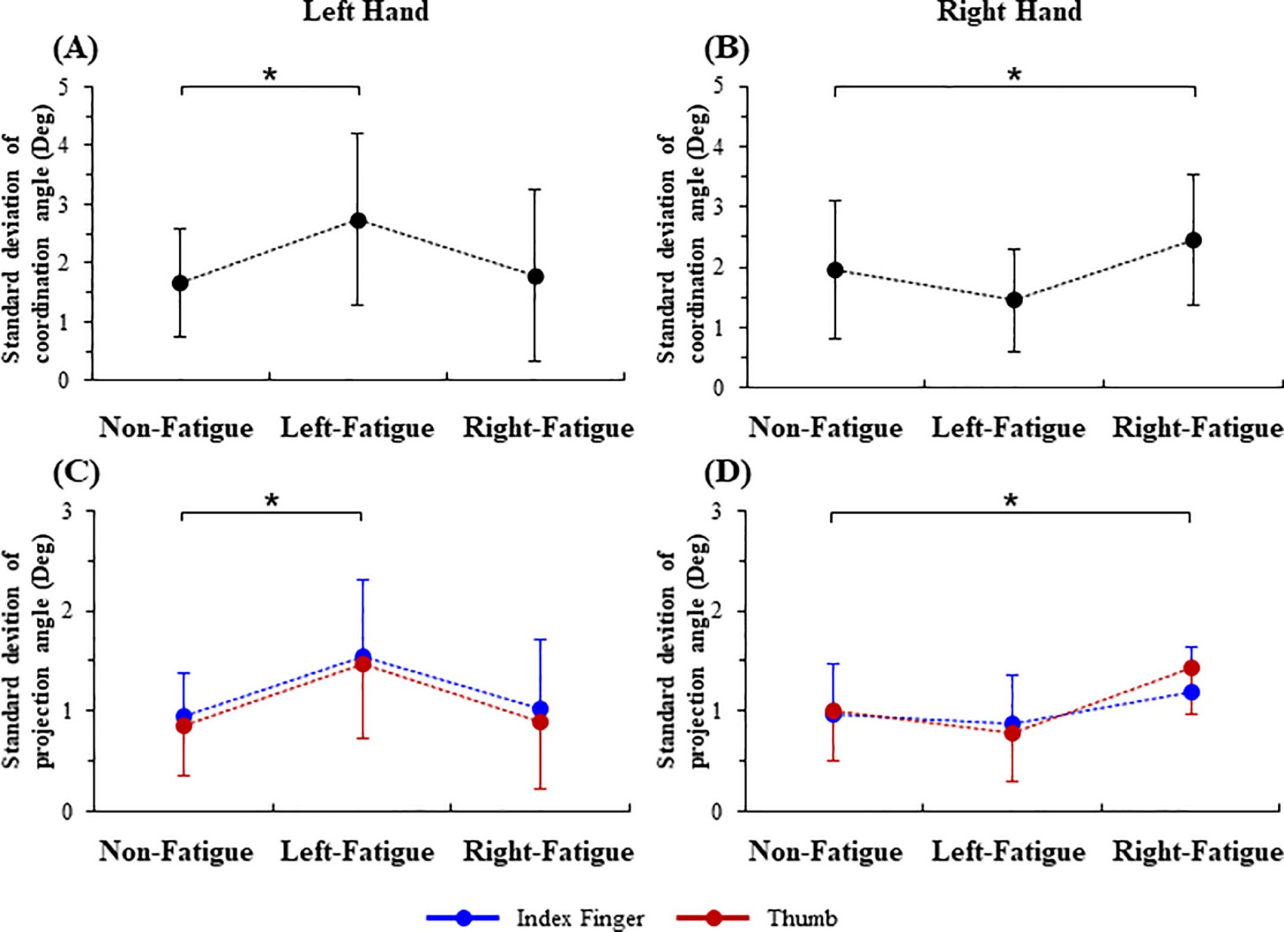
**Standard deviation of the coordination angle and projection angle under non-fatigue, left-fatigue and right-fatigue.** (A) and (B) standard deviation of the coordination angle for the left and right hands, respectively; (C) and (D) standard deviation of the projection angle for the left and right hands, respectively. * *p* < 0.05.

The standard deviation values of coordination angle and projection angle under the non-fatigue, left-fatigue and right-fatigue conditions were shown in Fig 5. The ANOVA tests showed significant main effects of fatigue conditions on the ipsilateral coordination angle across the two digits, and on the projection angle of both the thumb and index finger during the left- (coordination angle: *F*_1.31, 19.69_ = 4.40, *p* = 0.04, Fig 5A; projection angle: *F*_1.33, 19.94_ = 6.55, *p* = 0.01, Fig 5C) and right-hand precision grip (coordination angle: *F*_2,30_ = 6.70, *p* < 0.01, Fig 5B; projection angle: *F*_2,30_ = 5.81, *p* = 0.01, Fig 5D). The fatigue did not affect the contralateral standard deviation of coordination angle or projection angle. For example, there was no significant difference between the right-fatigue and non-fatigue conditions for the standard deviation of coordination angle (*p* = 0.59) or for the standard deviation of projection angle (*p* = 0.61) during the left-hand precision grip. On the other hand, no significant difference was observed between the left-fatigue and non-fatigue conditions for the standard deviation of coordination angle (*p* = 0.13) or for the standard deviation of projection angle (*p* = 0.32) during the right-hand precision grip. Moreover, there was no significant main effects of digits (thumb vs. index finger) on the standard deviation of projection angle during the left- (*p* = 0.14, Fig 5C) or the right-hand precision grip (*p* = 0.34, Fig 5D).

## Discussion

This study investigated the effects of muscle fatigue on the directional coordination of the force vectors of the thumb and index finger during precision grip. The force vector directions were quantified using the coordination angle and projection angle and were compared across the three fatigue conditions–the non-fatigue, left-fatigue and right-fatigue. Results showed that the unilateral hand muscle fatigue affect the amount and fluctuation of the ipsilateral coordination angle and projection angle for precision grip, but had little effects on the contralateral digit force directions for precision grip (Figs 4 and 5). These results confirmed the hypothesis that muscle fatigue may influence the inter-digit coordination of the force vector directions in dexterous manipulation particularly for the ipsilateral hand. It is noteworthy that fatigue did not influence the amount or variability of grip forces (Table 1), which confirms that the changes in the coordination angle and project angle with fatigue should not be attributed to the variations of grip force.

Results showed that the muscle fatigue significantly reduced the mean of the ipsilateral coordination angle during precision grip—the left coordination angle decreased from 150.13 ± 12.02° without fatigue to 142.49 ± 15.43° with left fatigue (Fig 4A); and the right coordination angle decreased from 151.19 ± 8.75°without fatigue to 145.03 ± 12.59° with right fatigue (Fig 4B). Previous study has reported that as subjects were producing pinch force upon a spatially fixed apparatus, the coordination angle of their thumb and index finger force vectors was about 150.8°, which is consistent with the coordination angle value we observed in the current study, during precision grip of a freely movable object without fatigue [15]. The reduced coordination angle with fatigue suggests higher deflection of force vectors with respect to the contact surface and lower normal force components relative to the shear force components. Considering the grip force–the average force perpendicular to the contact surface, and load force–the total vertical shear force, form the major part of the normal and shear forces, respectively, the reduced coordination angle with fatigue observed in the current study corroborates the viewpoint that muscle fatigue may lead to a decrease in the grip force to load force ratio [21]. Moreover, the mean value of projection angle indicated a directional alteration of the index finger’s force vector due to fatigue (Fig 4C and 4D). For the index finger, the left and right projection angles with fatigue were 68.24 ± 7.05° and 70.99 ± 6.26°, respectively, significantly lower than the projection angles without fatigue (left: 72.04 ± 5.89°, right: 73.76 ± 4.59°). The decline of projection angle resulting from hand muscle fatigue suggests a lower normal to shear force ratio and lower safety margin, which may increase the risk of object slippage [26]. Previous studies reported that the deviations of index finger force vectors from the perpendicular direction to the contact surface were exacerbated in stroke patients [17] and elderly people [27], suggesting lower capacity of controlling the force directions due to motor deficits associated with stroke and ageing. The current study further reveals that fatigue could lead to a larger deviation from the perpendicular direction to the contact surface (lower projection angle). This finding suggested that the changes in muscle activation patterns due to fatigue could deviate fingertip force vectors [13, 28, 29] and reduce the capacity of digit force control.

Additionally, results showed that the fatigue led to increases in both the standard deviation of coordination angle across the two digits and the projection angle of both the thumb and index finger for the ipsilateral hand (Fig 3G–3J, Fig 5). These results demonstrated that fatigue could result in not only greater deviations of force vectors but also higher instability of force vector directions during precision grip. The unstable force vector direction could be firstly attributed to an augment in force fluctuation with fatigue. Previous studies have found that the inherent force fluctuations are profoundly influenced by the structures (e.g., anatomical arrangement of nerves and muscles) and functions (e.g., cooperation of multiple muscles) of motor system [4]. Greater force angle variability may provide evidence that the subjects with fatigue had a diminished capacity of scale in their muscle activations and force generations to maintain consistent digit force directions and to stabilize their thumb-index finger coordination, thereby leading to a reduction in the accuracy and steadiness of precision grip. In addition, by investigating the precision grip performance of the elderly population, Parikh et al found a greater variability of moment-to-moment fluctuations in force direction in old adults during a key-slot task, and attributed this change to an age-related decline of sensorimotor function [19]. Similarly, in the current study, the greater variability of inter-digit force vector coordination (standard deviation of coordination angle) and of the individual digit force direction (standard deviation of projection angle) may also reflect a compromised sensorimotor integration associated with fatigue. Todd et al. reported a common disturbance of sensorimotor control with muscle fatigue accompanied by clumsiness, which indicates the CNS may not adapt to the altered relationships between neural output and force or sensory feedback due to fatigue [20]. The fatigue-related deterioration due to the reduction in mechanoelectrical transduction or even impaired central processing of sensory signals could impair the subjects’ ability to delivery proper motor commands to produce well-scaled forces on each digits and well-coordinated force vectors across the digits during precision grip.

Results also demonstrated some discrepancies in force directional control between the thumb and index finger during precision grip. No significant difference between the thumb and index finger in the mean of projection angle for the right hand (Fig 4D), which is consistent with the previous study showing that the thumb and index finger had similarly force vector directions when maintaining precision pinch force at 5 N [30]. By contrast, for the left hand, the mean of projection angle for the thumb was significantly higher than that of the index finger (Fig 4C), suggesting a higher normal-to-shear force ratio as the thumb applied force upon the contact surface on the non-dominant hand. This finding convinces us that the dominant and non-dominant hands have difference in coordinating the force vector directions of their respective thumb and index finger for dexterous manipulation [31]. Moreover, the effects of fatigue were observable in the force direction of index finger rather than in that of the thumb (Fig 4C). This finding suggests that the force directional control of index finger would be more vulnerable to the muscle fatigue than the thumb, which may be attributed to the differences in the anatomical and neural arrangement of the thumb and index finger [32]. For example, with more intrinsic muscles, the thumb could apply greater grasping strength and greater directional dexterity for precision pinch [28], thereby having more tolerance to the modified muscle activation patterns and showing more reliability of force directional control under muscle fatigue than the index finger.

Muscle fatigue includes both peripheral and central processes. Previous study has found that a central fatigue could systematically influence the motor performance of both left and right limbs. In the current study, the effects of fatigue was only observed in the ipsilateral rather than the contralateral precision grip performance, suggesting a localized rather than a systematical fatigue was evoked in this study. Another reason would be that the directional coordination of force vector examined in the current study was not sensitive enough to the potential central fatigue. It would be of interest to investigate whether the effects of fatigue could transfer from one hand to the other, effectively leading to changes in manipulative tasks, by either employing more effective fatigue tasks or applying more fatigue-sensitive parameters.

There would be some limitations in this study. During the experiment, subjects were required to hold the apparatus as stably as they could, maintaining the base of the apparatus parallel to the surface of testing table. The raters were supervising the subjects’ posture and the object’s orientation. Once the object showed obvious tilt, subjects were required to immediately stop this trial and were instructed for another trial with correct operation. However, because the orientation of the held object was not recorded during experiment, the potential effects of orientation on force vector directions cannot be fully excluded. Therefore, in the future study, the object’s orientation during precision grip would be under real-time supervision by a three-dimensional motion capture system. Based on the kinematic data, real-time orientation adjustment would be available and the potential effects of orientation on force vector direction could be minimized.

## Conclusion

This study investigated the effects of muscle fatigue on the directional coordination of thumb and index finger forces during precision grip. The muscle fatigue led to decreases in the mean of coordination angle across the thumb and index finger and the projection angle of the index finger in the ipsilateral hand. In addition, the fatigue induced increased variability of both the coordination angle across digits and the projection angle of individual digit in the ipsilateral hand. These results imply that the muscle fatigue could interfere with the grasping stability by altering the directional coordination of all the involved digits and the force direction control for each individual digit.
